# Phylogenetic Relationships among NE Atlantic Plocamionida Topsent (1927) (Porifera, Poecilosclerida): Under-Estimated Diversity in Reef Ecosystems

**DOI:** 10.1371/journal.pone.0016533

**Published:** 2011-02-09

**Authors:** Julie Reveillaud, Rob van Soest, Sofie Derycke, Bernard Picton, Annelien Rigaux, Ann Vanreusel

**Affiliations:** 1 Marine Biology Section, Biology Department, Ghent University, Ghent, Belgium; 2 CeMoFE, Center for Molecular Phylogeny and Evolution, Ghent, Belgium; 3 Zoological Museum of the University of Amsterdam (ZMA), Amsterdam, The Netherlands; 4 Department of Natural Sciences, National Museums Northern Ireland, Holywood, Northern Ireland, United Kingdom; Ecole Normale Supérieure de Lyon, France

## Abstract

**Background:**

Small and cryptic sponges associated with cold-water coral reefs are particularly numerous and challenging to identify, but their ecological and biochemical importance is likely to compete with megabenthic specimens.

**Methodology/Principal Findings:**

Here we use a combination of the standard M1M6 and I3M11 partitions of the COI fragment, partial rDNA 28S sequences and morphology to delineate small encrusting *Plocamionida* species. In total, 46 specimens were retrieved from seven shallow to deep-water coral locations, crossing 3,000 km along the European margins. Our work provides evidence that the *Plocamionida ambigua f. tylotata* and *f. grandichelata* can be considered valid species, whereas *Plocamionida ambigua f. tornata* corresponds to the species *P. ambigua*. Within the monophyletic group of *Plocamionida*, *P. microcionides* is shown as really divergent from the other taxa, and four putative new *Plocamionida* species are suggested.

**Conclusions/Significance:**

This study shows that the use of molecular and morphological information in an integrative approach is a powerful tool for the identification of sponge species, and suggests that an under-estimated biodiversity of sponges occurs in cold-water coral reefs.

## Introduction

Sponges represent one of the most remarkable groups in deep-water coral ecosystems [1], [2]. The high biodiversity and abundance of these filter-feeders (with a total of 191 species recorded in Irish bathyal coral reefs, [3]) contrasts with the paucity of the coral reef building species, predominantly *Lophelia pertusa* (Linnaeus 1758) and *Madrepora oculata* (Linnaeus 1758). Ecologists initially focused on large-sized, bright-coloured or conspicuous Porifera species (e.g. [4]–[6]), but the extensive presence of small sized and morphologically cryptic sponges in cold-water coral reefs (CWRs) has widely been noted [3], [7]–[10]. Single dead coral branches from cold waters can contain up to 15 sponge species [9]. The distribution of CWRs along the European margins has now been well documented [11], and several coral hotspots are found along the continental margin off Ireland, off Norway, and in the Mediterranean basin. Nevertheless, only few biodiversity studies have addressed the substantial diversity of deep-sea sponges associated with CWRs [3], [8]–[10]. Such lack of knowledge forms a substantial impediment for establishing baselines of biodiversity and for the efficient management of this group [9], which is of particular interest for the pharmaceutical industry [12]. Moreover, the significance of these deep and nutrient rich hotspot ecosystems for potential centers of endemism has direct implications for both regional diversity and local endemicity.

The sponge genus *Plocamionida* Topsent (1927) (Class Demospongiae, Order Poecilosclerida, Family Hymedesmiidae) is widely distributed along the continental margins of Europe, and occurs from the Mediterranean Sea and the Azores to “high latitudes” in the NE Atlantic [9], [13]–[15]. Within CWR environments, *Plocamionida* species encrust rocks or hard corals in thin (<5 mm) sheets of brown coloration and can be locally abundant. Occasionally, shallow-water occurrence is reported, but their main occurrence is at depths of 50 m and deeper. Although the genus has excellent morphological markers, the taxonomic distinctness of its European species remains highly contentious. Two species names, *P. ambigua* (Bowerbank, 1866) and *P. microcionides* (Carter, 1876) have been considered as valid separate species, or as synonyms of a single variable species (e.g. [14], [15]). Furthermore, a number of ‘formas’ have been proposed by various authors for specimens with deviating spicule characteristics, *Plocamionida microcionides f. achelata* Topsent, 1928, *Plocamionida ambigua f. grandichelata* Brøndsted, 1932, *Plocamionida ambigua f. tornotata* Brøndsted, 1932 and *Plocamionida ambigua f. tylotata* Brøndsted, 1932. The latter form has been given species status by Alander [16] and Picton and Goodwin [17]. *Plocamionida* remains a group of sponges that are notoriously difficult to identify because the intra- and interspecific character variation is not well understood, and has given rise to disagreements between taxonomic experts.

The first aim of this study is to investigate the phylogenetic relationships of *Plocamionida* species and formas from the Gulf of Cadiz to Norway occurring in CWRs. Specimens from one shallow water population were also included. We used phylogenetic congruence criteria between the cytochrome oxidase c subunit I (COI) and the independent nuclear region D3–D5 of the rDNA 28S gene to delineate evolutionary significant units, and to reveal the presence of cryptic species within the studied material. The standard (COI) barcoding fragment, amplified with the universal primers of Folmer et al. ([18]; hereafter called the M1M6 partition) is generally too conserved in diploblast phyla [19] and has led to some difficulties in resolving taxonomic and phylogeographic relationships in sponges [20], [21]. On the other hand, genetic studies above and below the species level have been performed using this COI partition [10], [22]–[25]. In addition, the COI downstream I3M11 partition showed more resolution than the standard M1M6 partition [26]. It proved useful at interspecific level [27] and to determine the genetic population structure of Caribbean and European sponge species [28], [29]. Herein, we seize the opportunity to compare the I3M11 partition to the M1M6 partition in terms of amplification success and substitution pattern, as well as to assess the potential of their combination for species level delineation. Second, the phylogenetic units were morphologically analyzed to investigate whether concordant molecular lineages are also morphologically distinct and to resolve the current taxonomic difficulties in *Plocamionida*.

## Materials and Methods

### Sampling

**Figure 1:**
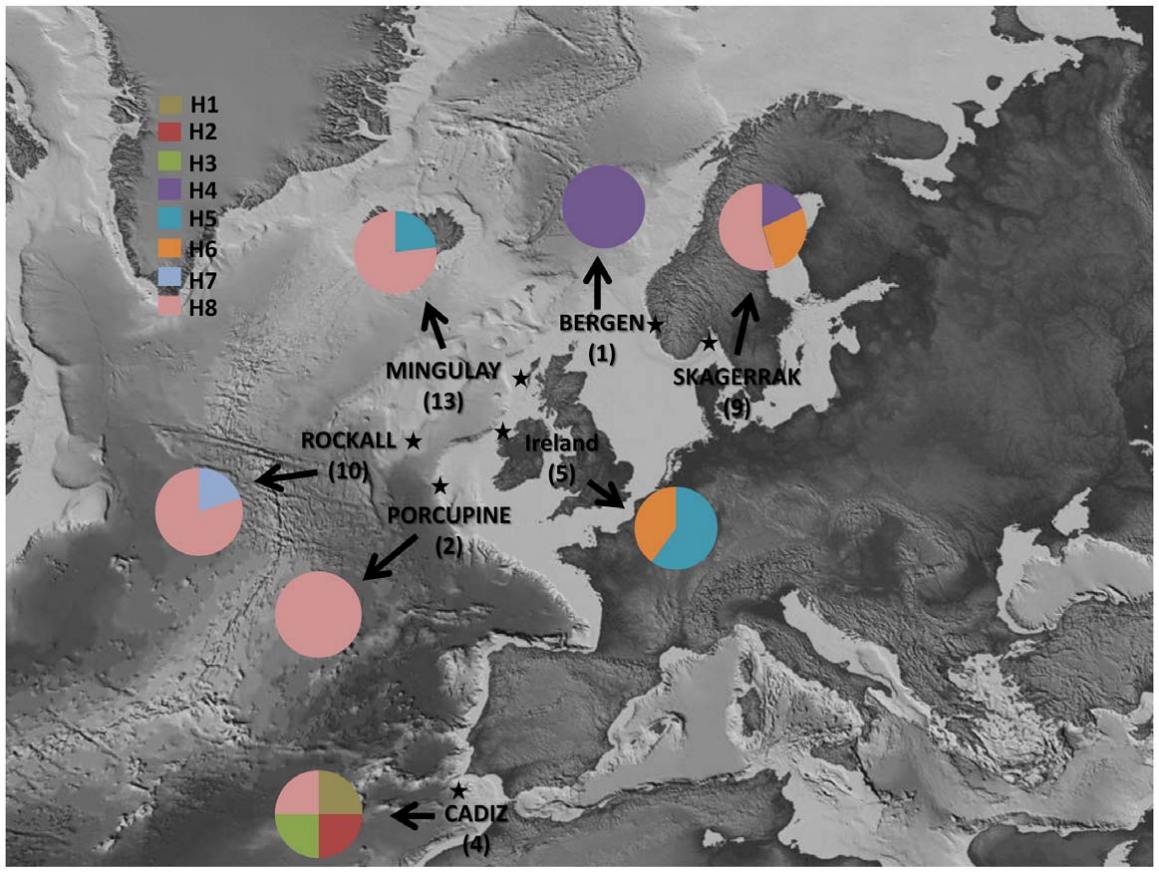
Map showing sampling locations of *Plocamionida* species (numbers in parenthesis indicate sample sizes) and geographical distribution of ESUs. Map was provided by the project Hotspot Ecosystem Research and Man's Impact on European Seas (HERMIONE). Sampling location are given in uppercase letters for deep-water samples (>50 m) and in lowercase letters for shallow-water samples.

A total of 46 specimens of *Plocamionida* were collected from seven locations along the Atlantic continental margin spanning about 3,000 km (Fig. 1). Normal storm waves disturb the seafloor significantly down to 50 m [30] and this depth was used to separate shallow from deep-water environments. Deep-water specimens (>50 m) were collected with boxcores or with a Remote Operated Vehicle (ROV) during five cruises and one specimen was dredged up from the coast of Norway in Bergen (Table S1). Shallow-water specimens were collected by scuba diving at The Maidens, Northern Ireland. Specimens were detected by searching dead coral branches and stones using a low power microscope. All samples were preserved in absolute ethanol and stored at room temperature until further processing. *Plocamionida*-like individuals were identified by looking at their skeletal structure using thick sections air-dried on microscopical slides and mounted in Canada balsam. Voucher specimens are deposited in the Porifera collection of the Zoological Museum of Amsterdam (ZMAPOR) and in the Ulster Museum, Belfast (BELUM) and are available upon request. The list of studied species and localities with their abbreviations is given in Table S1.

### DNA extraction, PCR setup and amplifications

DNA extraction from samples was performed using the DNeasy Blood and Tissue Kit (Qiagen) in accordance with the manufacturer's instructions. Polymerase chain reaction (PCR) amplifications were carried out in a total volume of 47 µl, with 5 µl of 10 x PCR buffer (Qiagen), 5 µl of 10x CoralLoad (Qiagen), 1 µl MgCl2 (25 mM), 1 µl dNTP (10 mM each), 0.5 µl of BSA (10 µg/µl), 1 µl of forward and reverse primer (25 µM), 0.25 µl TopTaq DNA polymerase (5 u/µl, Qiagen), 1 µl of template DNA and 31.25 µl of HPLC grade water.

A∼1200 bp long fragment of the cytochrome c oxidase subunit I (COI) mtDNA gene was initially amplified from ten random specimens using the universal primer LCO1490 [18] and the reverse primer COX1-R1 (5′-TGTTGRGGGAAAAARGTTAAATT -3′) [31]. Polymerase chain reaction (PCR) cycling conditions included an initial denaturation step of 5 min at 94°C, 5 cycles (94°C for 1 min, 48°C for 1 min and 72°C for 1 min 30), 30 cycles (94°C for 1 min, 50°C for 1 min and 72°C for 1 min 30) and a final extension at 72°C for 10 min. Based on these ten sequences, specific *Plocamionida* sp. primer COIPlo20F (5′-GCTTTTGCGGGGATGATAGGTAC-3′) and COI800Rev (5′-TCTACATCCATTCCTACTGTAAACATGTG -3′) were developed to amplify the M1M6 partition ([18]) under a temperature regime of 5 min at 94°C followed by 35 cycles of 94°C for 45 s, 47°C for 45 s, 72°C for 45 s and a final extension at 72°C for 10 min. PCR amplifications of the I3M11 partition were performed using the primers COI800Fwd (5′- CACATGTTTACAGTAGGAATGGATGTAGA-3′) (reverse complement from the specific primer COI800Rev) and COX1-R1 under a temperature regime of 94°C for 2 min, followed by 30 cycles of (94°C for 30 s, 47°C for 30 s, 72°C for 30 s) and a final extension at 72°C for 10 min.

The D3–D5 fragment of the rDNA 28S gene fragment was amplified using the primers RD3A 5′-GAC CCG TCT TGA AAC ACG A-3′ and RD5B2 5′- ACA CAC TCC TTA GCG GA-3′ [32] under a temperature regime described in Reveillaud et al. [10].

### PCR product processing and sequencing

The PCR-amplified products were loaded onto a 1% agarose gel, checked for size, and sequenced in both directions through a Perkin- 234 Elmer ABI 3130 capillary DNA sequencer. The PCR products were purified by incubation at 37°C using exonuclease I, *E. coli* (20 U µl^−1^; Fermentas) and FastAP thermosensitive alkaline phosphatase (1 U µl^−1^; Fermentas), and labelled using the Big Dye Terminator v3.1 Cycle Sequencing Kit (Applied Biosystems). Chromatograms obtained from the automated sequencer were read and contigs assembled using the sequence editing software SeqMan Pro v.7.1.0 (DNASTAR Lasergene). We checked the poriferan origin of the sequences by BLAST searches against the Genbank database (http://www.ncbi.nlm.nih.gov/BLAST/) and their relationship to other taxa in a phylogenetic tree as described in Erpenbeck et al. [22]. All the sequences are deposited in the European Molecular Biology Laboratory (EMBL) under accession numbers FR687219–687251.

### Sequence alignment and phylogenetic analyses

COI and 28S sequences were aligned using the web interface of the multiple alignment software MAFFT ([33]; available at http://www.ebi.ac.uk/Tools/mafft/index.html), under default settings. Ambiguous positions in the D3–D5 region of 28S were discarded. Our own sequences of the poecilosclerid sponges *Desmacella inornata* (Family Desmacellidae) and *Mycale lingua* (Family Mycalidae) were used as uniform outgroup for all analyses.

Both partitions of the COI gene were combined for phylogenetic analyses. The COI and the 28S partitions were separately analyzed and then combined for the same set of specimens whenever possible. A partition homogeneity test performed in PAUP* 4.0b10 [34] with 100 replicates between the COI and the 28S datasets showed that data partitions were not significantly incongruent (p = 1). Phylogenetic reconstructions of the nucleotide data sets were performed using the maximum likelihood (ML) criterion of PAUP* 4.0b10 [34] and Bayesian inference (BI) criterion of MrBayes 3.1.2 [35]. We used Modeltest 3.06 [36] as well as its simplified version MrModeltest 1.1 [37] to estimate the best-fitting nucleotide model under the Akaike Information Criterion (AIC) for each independent gene for the ML and the BI analysis respectively. The GTR+I best fitted the COI data set for both ML and BI reconstructions whereas for the 28S dataset, the models selected by AIC were TrN+I for the ML reconstructions and HKY+I for the BI analyses. The GTR+G+I model was selected for ML and BI for the combined COI and 28S dataset. ML trees were calculated using heuristic searches and a tree bisection and reconnection (TBR) branch swapping algorithm (10 000 rearrangements), and a random stepwise addition of sequences in 100 replicate trials. Nodal support was estimated with a bootstrap procedure with 100 replications and 10 replicate trials of sequence addition. Bootstrap supports (BS)>70 were considered high enough to support clades in ML reconstructions. Bayesian inference analyses were performed with four Markov-chains for each gene. For COI and the combined COI-28S dataset, analyses were performed with 1 million generations sampled every 1000 generations while 300000 generations sampled every 300 generations were used for the 28S dataset. After all analyses, the average standard deviation of split frequencies was below 0.01. We used the burn-in value of 25%. In BI reconstructions, posterior probabilities (PP)>95 were considered to support clades.

Maximum intraclade (whenever more than one haplotype was found) and minimum interclade/branch corrected p-distances were calculated for the COI and the 28S gene fragment using PAUP* 4.0b10 [34] under the respective models from the ML analyses.

### Morphological analyses

Microscopic examination of spicule ornamentations and measurements of spicule micrometries were done using a compound Leitz microscope at 10×10× and 10×40× magnifications, on dissociated spicule mounts obtained after boiling a fragment in concentrated nitric acid, mounted in Canada balsam. All specimens were examined and classified using the following morphological criteria: presence or absence of spines on the blunt ends of the choanosomal large styles, the tylote, mucronate or pointed shape of the tornote endings, the simple or compound shape of the spines in the acanthostrongyles, and size of the chelae (more or less than 30 microns). For the latter character, at least 25 chelae spicules were measured in each preparation and no overlap was found between the ranges 15–25 versus 30+ µm.

## Results

### Phylogenetic reconstruction

#### mtDNA COI dataset

**Figure 2:**
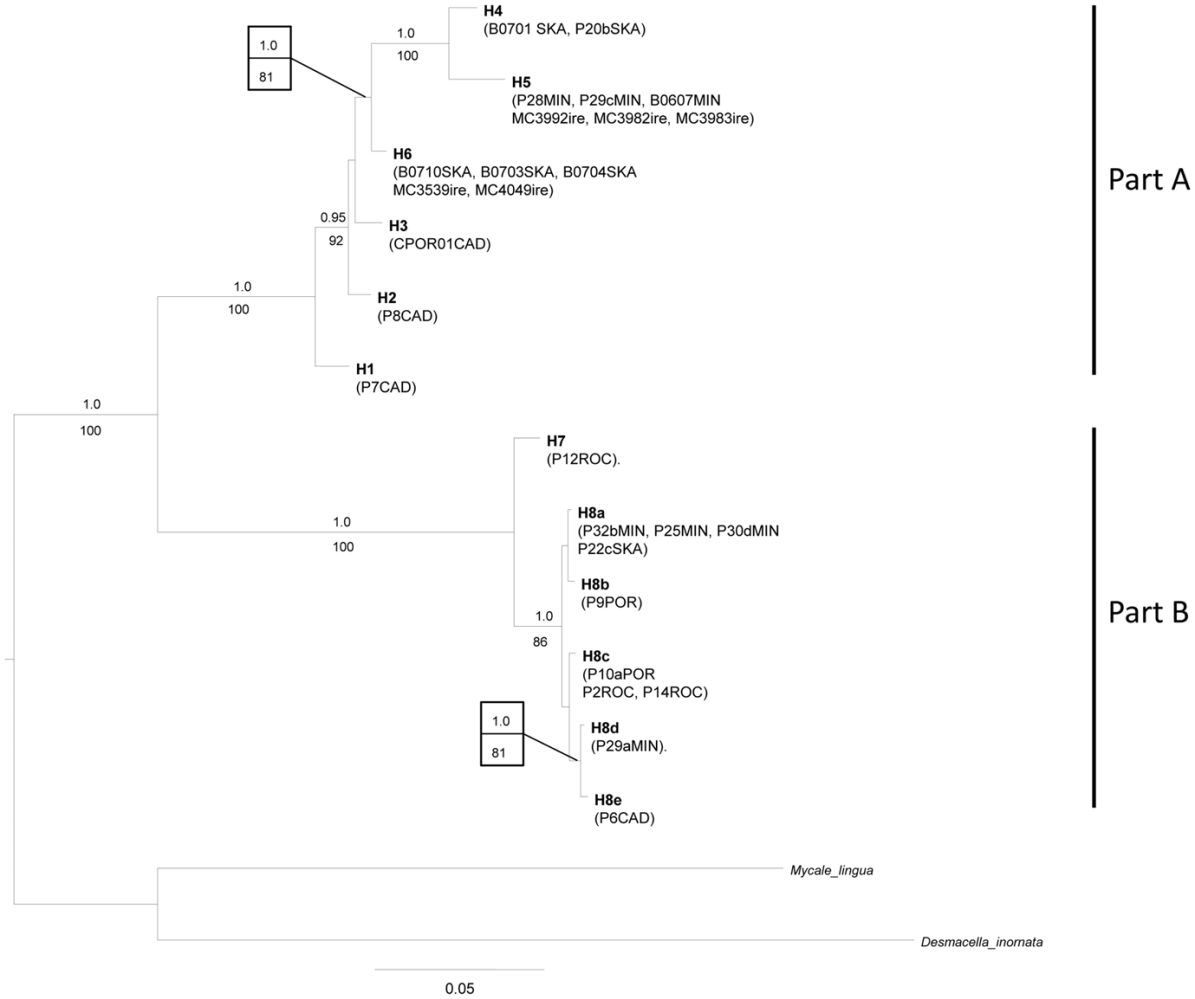
Bayesian majority-rule consensus tree of the mtDNA COI M1M6 and I3M11 partitions. Bayesian posterior probabilities (when >0.95) and the ML bootstrap values (when >70) are indicated above and below branches, respectively. For information on the specimens (listed in parenthesis) see Table S1.

The resulting data sets comprised 27 specimens and 12 haplotypes with 777 characters (88 parsimony informative, ts/tv ratio  = 1.941) for the M1M6 partition and 46 specimens, 13 haplotypes with 363 nucleotides (53 parsimony informative, ts/tv ratio = 1.65) for the I3M11 partition (Table S1). Both partitions could unambiguously be aligned and translated into respectively a functional 259 and 121 amino-acid protein sequence of the COI. No frame shifts or point mutations were present. The success rate of the COI amplification in all specimens varied along the COI fragment, with 58% in the M1M6 partition and 85% in the I3M11 partition. The combined dataset contained only specimens from which both partitions were obtained. It comprised 27 specimens and 12 haplotypes with 1140 characters (138 parsimony informative, ts/tv ratio  = 1.685) and was used for the phylogenetic reconstruction of the COI gene (Fig. 2).

*Plocamionida* specimens formed a well supported monophyletic group, with high Bayesian PP and ML BS (1.0/100). The COI tree was divided into two well supported parts A and B (1.0/100) separated by high genetic divergence values (GTR+I corrected p-distance of 14.5% to 20%, Table 1). Part A showed six highly divergent haplotypes (*H1*–*H6*) with GTR+I corrected p-distance ranging from 1.3 to 5.9%. *H4* and *H5* were more closely related to each other than to any of the other sequences. In addition *H4*–*6* and *H2*–*6* formed well supported subclades (1.0/81 and 0.95/92 respectively). Genetic divergence between the six sequences of Part B was much lower (0.5% to 1.9%) than those within part A. Part B was substructured into a well supported clade (*H8a*–*e*; 1.0/86) and a highly divergent single sequence (*H7*). The sequences *H8d*–*e* were found to be more closely related to each other than to the other *H8* haplotypes.

**Table 1:** mtDNA genetic divergence values between and within Evolutionary Significant Unit (ESU).

COI	H7	H8a–e	H4	H5	H6	H1	H2	H3
**H7**	-							
**H8a**–**e**	0,019	0,005						
**H4**	0,188	0,184	-					
**H5**	0,197	0,200	0,023	-				
**H6**	0,165	0,161	0,032	0,040	-			
**H1**	0,148	0,149	0,055	0,059	0,024	-		
**H2**	0,147	0,145	0,039	0,042	0,013	0,022	-	
**H3**	0,155	0,155	0,040	0,045	0,014	0,023	0,014	-

The COI genetic divergence (corrected p-distance) between ESU are provided below diagonal and between individuals within ESU on diagonal. The different haplotypes are presented in Fig. 2.

#### rDNA 28S dataset

**Figure 3:**
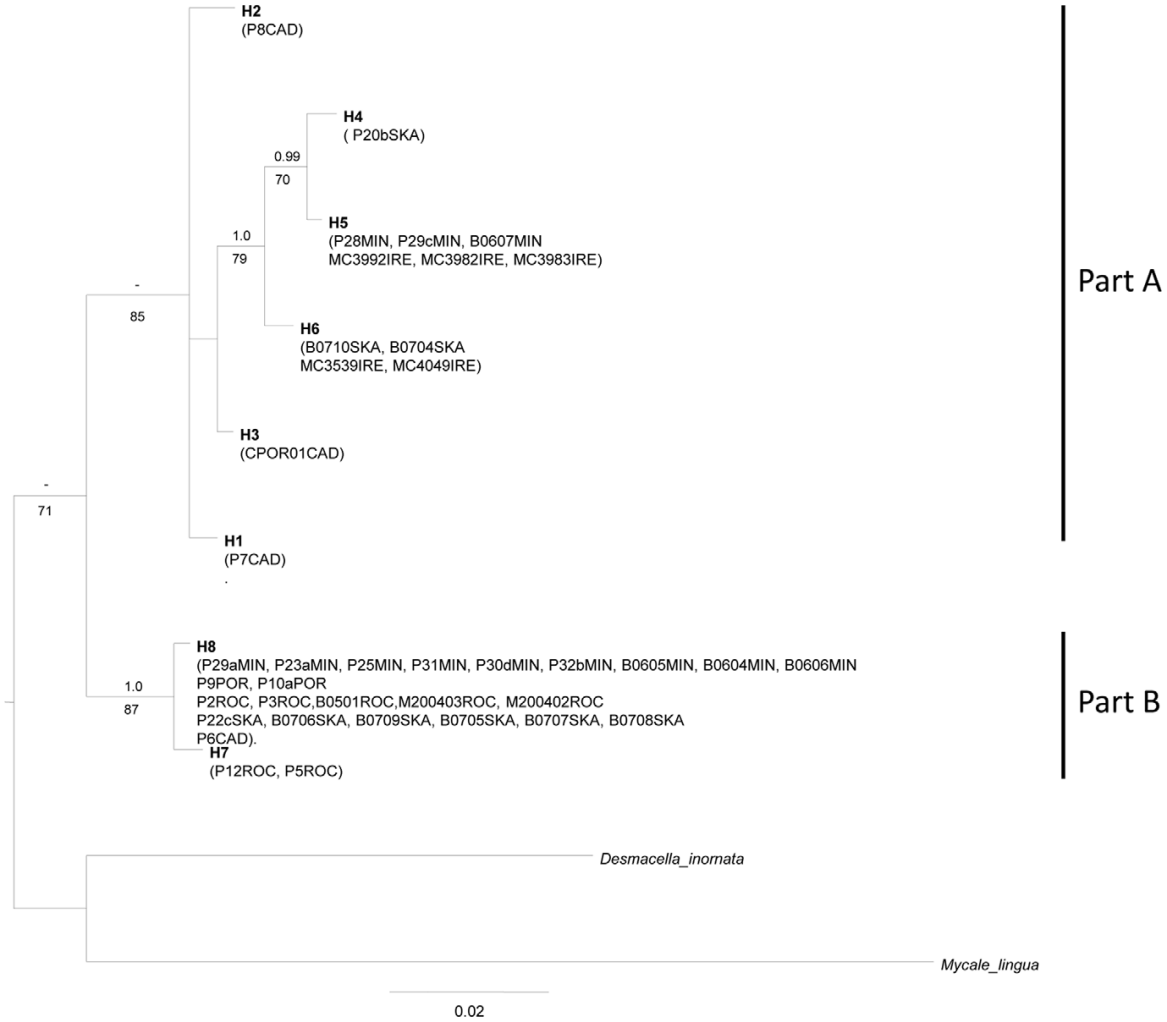
Bayesian majority-rule consensus tree of the rDNA 28S fragment. Bayesian posterior probabilities (when >0.95) and the ML bootstrap values (when >70) are indicated above and below branches, respectively. For information on the specimens (listed in parenthesis) see Table S1.

The resulting data sets comprised 39 specimens and 8 genotypes with 620 nucleotides (15 parsimony informative). Phylogenetic relationships using the 28S fragment were highly similar to the ones obtained from COI, but received less support (Fig. 3): *Plocamionida* specimens were recovered as a monophyletic group (-/71) and the deeper parts A and B were recovered (-/85 and 1.0/87, respectively). The subclades *H4*–*5* and *H4*–*6* were recovered with high support (0.99/70 and 1.0/79, respectively).The different haplotypes identified in COI as *H8a*–*e* shared a single 28S sequence (*H8*), with a sequence divergence of 0.1% from *H7* (Table 2).

**Table 2:** 28S genetic divergence values between and within Evolutionary Significant Unit (ESU).

28S	H7	H8	H4	H5	H6	H1	H2	H3
**H7**	-							
**H8**	0,001	-						
**H4**	0,033	0,031	-					
**H5**	0,028	0,026	0,001	-				
**H6**	0,026	0,024	0,007	0,004	-			
**H1**	0,020	0,019	0,012	0,010	0,008	-		
**H2**	0,023	0,021	0,012	0,010	0,010	0,005	-	
**H3**	0,020	0,019	0,009	0,006	0,005	0,003	0,005	-

The 28S genetic divergence (corrected p-distance) between ESU are provided below diagonal and between individuals within ESU on diagonal. The different haplotypes are presented in Fig. 3

Sequence divergence ranged from 1.9 to 3.3% between part A and B, and was 1.2% and 0.1% within Part A and B, respectively (Table 2).

#### Combined dataset COI-28S

**Figure 4:**
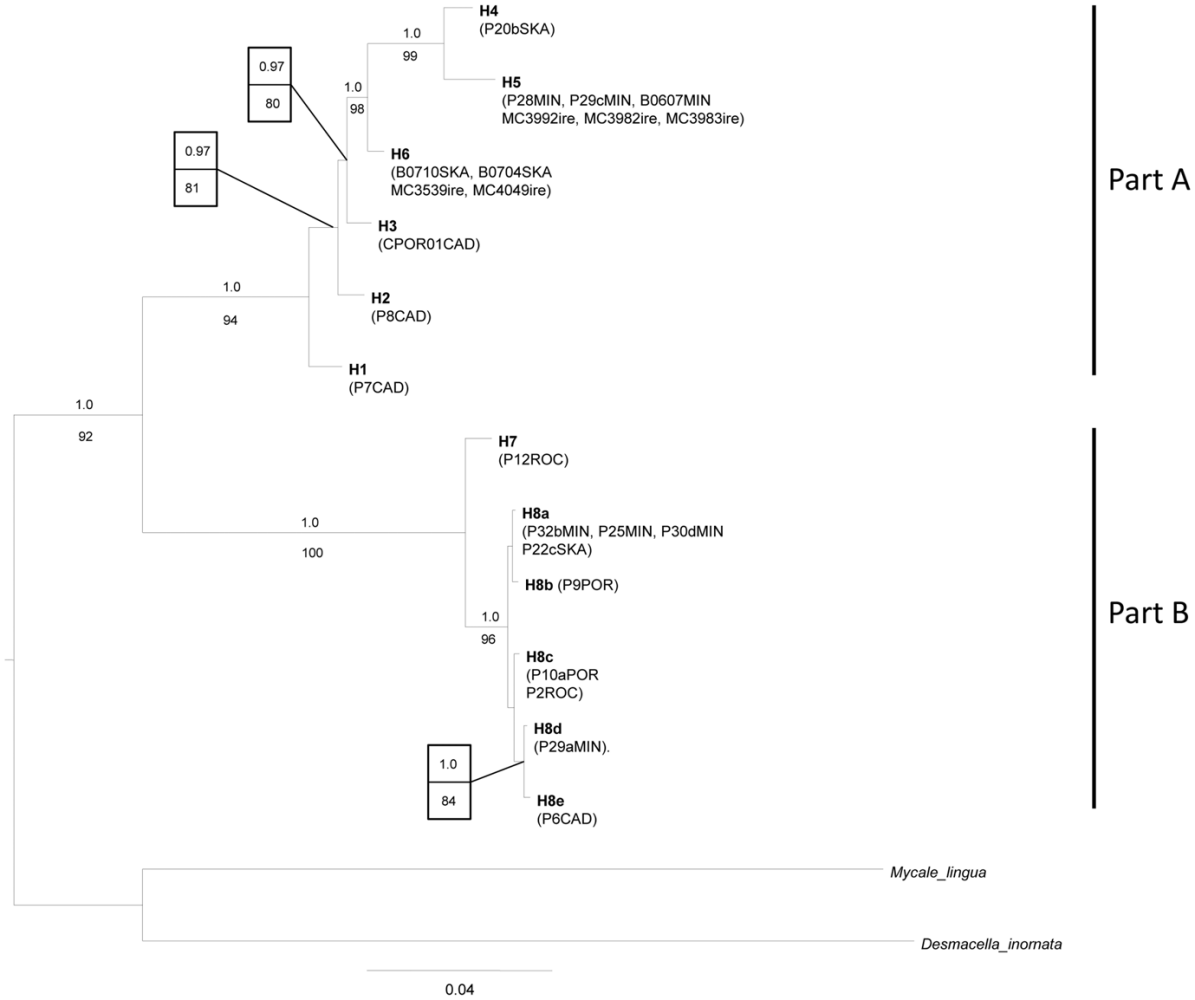
Bayesian majority-rule consensus tree of the concatenated dataset (COI-28S). Bayesian posterior probabilities (when >0.95) and the ML bootstrap values (when >70) are indicated above and below branches, respectively. For information on the specimens (listed in parenthesis) see Table S1.

The concatenated COI-28S dataset comprised 24 specimens with 1760 characters. The monophyly of the *Plocamionida* specimens was highly supported (1.0/92), and Parts A and B were again recovered with high support (1.0/94 and 1.0/100, respectively; Fig. 4). *H4* and *H5* were found more closely related to each other as found in COI and subclades *H4*–*6* and *H2*–*6* were highly supported (1.0/98 and 0.97/81 respectively). Subclade *H8a*–*e* within part B was recovered with high support (1.0/96) and *H8d*–*e* were found to be more closely related to each other, as previously found in COI. Based on the phylogenetic congruence between COI and 28S and the higher genetic divergence value between than within *H1*–*8* in both genes, we consider hereafter *H1*–*8* as independent Evolutionarily Significant Units (ESU).

### Morphological analysis

Most ESUs were characterized by morphological differences, except *H1*–*H3* and *H7*–*H8* (Table 3). Specimens from *H6* were characterized by spined large styles, tylote tornotes, a simple shape of the spines in the acanthostrongyle and the size of the chela being smaller than 30 microns (µm). In contrast, *H4* and *H5* specimens were characterized by a mucronate shape of the tornote endings. The size of the chela further differentiated *H4* and *H5* specimens. As mentioned above, the three Gulf of Cadiz specimens (*H1*, *H2* and *H3*) were morphologically similar and possessed the tylote tornotes in combination with a large chela >30 µm. Part B specimens possessed smooth large styles, fusiform tornotes, a truncate shape of the spines in the acanthostrongyle and a chela smaller than 30 µm. The only exception was *H8e* (P6) from the Gulf of Cadiz which had a chela >30 µm.

**Table 3:** Morphological characters of *Plocamionida* specimens.

Sample	Localities Abb.	large style	tornotes	acanthostrongyle	chela	Identity
**M2004-02**	**ROC**	**smooth**	**fusiform**	**truncate**	**<30**	**H8**
**M2004-03**	**ROC**	**smooth**	**fusiform**	**truncate**	**<30**	**H8**
**P2**	**ROC**	**smooth**	**fusiform**	**truncate**	**<30**	**H8**
**P3**	**ROC**	**smooth**	**fusiform**	**truncate**	**<30**	**H8**
**P4**	**ROC**	**smooth**	**fusiform**	**truncate**	**<30**	**H8**
**P11**	**ROC**	**smooth**	**fusiform**	**truncate**	**<30**	**H8**
**P14**	**ROC**	**smooth**	**fusiform**	**truncate**	**<30**	**H8**
**B05-01**	**ROC**	**smooth**	**fusiform**	**truncate**	**<30**	**H8**
**P9**	**POR**	**smooth**	**fusiform**	**truncate**	**<30**	**H8**
**P10 a**	**POR**	**smooth**	**fusiform**	**truncate**	**<30**	**H8**
**P23 a**	**MIN**	**smooth**	**fusiform**	**truncate**	**<30**	**H8**
**P25**	**MIN**	**smooth**	**fusiform**	**truncate**	**<30**	**H8**
**P29a**	**MIN**	**smooth**	**fusiform**	**truncate**	**<30**	**H8**
**P30d**	**MIN**	**smooth**	**fusiform**	**truncate**	**<30**	**H8**
**P31**	**MIN**	**smooth**	**fusiform**	**truncate**	**<30**	**H8**
**P32b**	**MIN**	**smooth**	**fusiform**	**truncate**	**<30**	**H8**
**B06-03**	**MIN**	**smooth**	**fusiform**	**truncate**	**<30**	**H8**
**B06-04**	**MIN**	**smooth**	**fusiform**	**truncate**	**<30**	**H8**
**B06-05**	**MIN**	**smooth**	**fusiform**	**truncate**	**<30**	**H8**
**B06-06**	**MIN**	**smooth**	**fusiform**	**truncate**	**<30**	**H8**
**B07-05**	**SKA**	**smooth**	**fusiform**	**truncate**	**<30**	**H8**
**B07-06**	**SKA**	**smooth**	**fusiform**	**truncate**	**<30**	**H8**
**B07-07**	**SKA**	**smooth**	**fusiform**	**truncate**	**<30**	**H8**
**B07-08**	**SKA**	**smooth**	**fusiform**	**truncate**	**<30**	**H8**
**B07-09**	**SKA**	**smooth**	**fusiform**	**truncate**	**<30**	**H8**
**P22c**	**SKA**	**smooth**	**fusiform**	**truncate**	**<30**	**H8**
**P6**	**CAD**	**smooth**	**fusiform**	**truncate**	**>30**	**H8**
**P5**	**ROC**	**smooth**	**fusiform**	**truncate**	**<30**	**H7**
**P12**	**ROC**	**smooth**	**fusiform**	**truncate**	**<30**	**H7**
B07-03	SKA	spined	tylote	simple shape	<30	H6
B07-04	SKA	spined	tylote	simple shape	<30	H6
B07-10	SKA	spined	tylote	simple shape	<30	H6
MC3539	ire	spined	tylote	simple shape	<30	H6
MC4049	ire	spined	tylote	simple shape	<30	H6
P28	MIN	spined	mucronate	simple shape	<30	H5
P29c	MIN	spined	mucronate	simple shape	<30	H5
B06-07	MIN	spined	mucronate	simple shape	<30	H5
MC3992	ire	spined	mucronate	simple shape	<30	H5
MC3982	ire	spined	mucronate	simple shape	<30	H5
MC3983	ire	spined	mucronate	simple shape	<30	H5
P20b	SKA	spined	mucronate	simple shape	>30	H4
B07-01	SKA	spined	mucronate	simple shape	>30	H4
BER82-01	BER	spined	mucronate	simple shape	>30	H4
CPOR08-01	CAD	spined	tylote	simple shape	>30	H3
P8	CAD	spined	tylote	simple shape	>30	H2
P7	CAD	spined	tylote	simple shape	>30	H1

Specimens, their localities abbreviation (Abb.) as in Table S1, examined for the following morphological criteria: presence or absence of spines on the blunt ends of the choanosomal large styles, the tylote, mucronate or pointed shape of the tornote endings, the simple or compound shape of the spines in the acanthostrongyles, and size of the chelae (more or less than 30 microns). Their identity is provided by their corresponding Evolutionary Significant Unit (ESU). Part B's specimens in bold.

## Discussion

### Integrative Taxonomy in *Plocamionida*

The genetic results conform well to previous morphological proposals for the subdivision of *Plocamionida* into several distinct European taxa. The specimens are divided into eight ESUs, which were grouped into two clades: Part A (*H1*–*6*) and part B (*H7*–*8*) specimens were separated by high divergence values in the COI and 28S fragments (minimum corrected p-distance of 14.5% and 1.9% respectively) and showed consistent morphological differences (spined large styles in Part A specimens vs. smooth large styles in Part B specimens). Comparing the limited number of morphological characters against a robust and comprehensive phylogenetic (DNA) tree approved to be a fruitful approach for integrating the strengths of morphological data with those of sequence data.

Part A contains *Plocamionida ambigua* s.l. and the forms described by Brøndsted [38] as *f. tornotata*, *f. tylotata* and *f. grandichelata*. Specimens of ESU *H5* possess the characters described by Bowerbank [39] for the type specimen of *Plocamionida ambigua* and by Brøndsted [38] as *f. tornotata*. Haplotype *H6* specimens possess the characters described by Brøndsted [38] as *f. tylotata* and haplotype *H4* specimens possess the characters described by Brøndsted [38] as *f. grandichelata*. Given these morphological differences and the high genetic distance between *H4* and *H6* specimens (corrected p-distance of 3.2% in COI and 0.7% in 28S), as well as between *H4* and *H5* (corrected p-distance of 2.3% in COI and 0.1% in 28S), *f. tylotata* and *f. grandichelata* can be considered valid species, whereas *f. tornata* corresponds to the species *P. ambigua*. Three Gulf of Cadiz specimens of Part A (*H1*–3) appear to have deviating characters from the other *Plocamionida* specimens (spined large styles, tylote tornotes, simple shape of the spines in the acanthostrongyle and large chelae). This observation, in addition to the presence of unique haplotypes, divergent from the most closely related species (*P.tylotata*, *H6*) by p-distance values from 1.3% to 2.4% in COI and from 0.5% to 1% in 28S suggests that these specimens may actually be undescribed *Plocamionida* species. However, no morphological characters could distinguish the three different specimens from each other. Evidently, a larger number of specimens from these ESUs need to be analyzed to infer whether they may form a cryptic species complex.

Part B conforms almost entirely with the description of *Plocamionida microcionides* (Carter, 1876) as redescribed by Stephens [14]. Only the above mentioned H8e from the Gulf of Cadiz deviates by having large chelae, a characteristic used so far as critical to delineate species, in combination with molecular data. This one COI sequence indicates that the classification of species purely by means of morphology may be difficult and that the size of the chelae within *Plocamionida* species can be an ambiguous diagnostic character in some *Plocamionida* species. It again emphasizes the need to study morphological variation in combination with other data, such as genetic variation. In addition, *H7* is highly divergent from the other *P. microcionides* specimens in the molecular analyses of COI and 28S (p-distance values of 1.9% and 0.1% respectively) and the combined partitions, but its morphological features are identical to the ones of Part B. This may indicate that *P. microcionides* is actually a species complex. However, more specimens of *H7* are required to support this hypothesis. Additional investigations (behavioural, ecological, etc.) and further taxonomic analyses (cytology, chemistry) might also be needed.

The high and non-overlapping genetic divergence values among ESUs (from 1.3% to 20%), and within the more widespread and genetically diverse species *H8* (0.5%, Table 1) indicates the usefulness of the COI partitions for the molecular distinction of species in our data set. A slightly lower ratio of transition/transversions (1.45 vs. 1.941) was observed in I3M11 vs. M1M6 partitions when using the 27 specimens for which both M1M6 and I3M11 sequences were available. It confirms the more progressive stage in character evolution of the I3M11 partition compared to the M1M6 partition [26]. Moreover, the I3M11 partition was much easier to amplify than the M1M6 partition. Sequences of the M1M6 partition were more often impeded by contaminations (hydrozoans, microbial symbionts, etc.). Our study further confirms the resolution power and suitability of the I3M11 COI partition for low level phylogenies such as barcoding, (Sponge Barcoding project,), as the same number of ESU (eight) was detected using the M1M6 and I3M11 partitions separately or even jointly. Although the low COI genetic differences between some of the ESUs (Table 1:1.3 to 1.9%) are clearly smaller than the interspecific distances found within other genera, such as *Hexadella* (Order Verongida, 3.9 to 8.7% [10]) or *Scopalina* (Order Halichondrida, 11 to 22% [25]), Poppe et al. [40] reported very low genetic distance values (maximum 1.8%) between morphologically distinct *Psammocinia* species (Order Dictyoceratida). Consequently, the COI marker seems to show different levels of genetic variation between different sponge taxa. The 28S tree showed major congruences with the COI tree, although the number of highly supported clades recovered in 28S was lower. This marker, in combination with COI, was found suitable to highlight putative cryptic species within *Plocamionida*, such as *H1*–*3* and *H7*–*8*. The combination between molecular data and morphological characters proved useful for differentiating *Plocamionida* species and for establishing their phylogenetic relationships. All trees showed the same topology, which confirms the consistency of the arrangement. This study reinforces the utility of integrative taxonomy [25], [41]–[43].

### Bathymetric and geographic distribution of *Plocamionida* species

*P.tylotata*, and *P.ambigua* were shown to present a wide bathymetric range distribution, with records in both deep (Skagerrak and Mingulay CWRs respectively), and shallow water (the Irish locality), while *P.microcionides* and *P.grandichelata* were found only in deeper habitats (≥50 m; [14], [38], [44] this study). So far, *P.grandichelata* was only reported from Scandinavian waters (the Faroe, [38]; CWRs area from Bergen and Skagerrak, this study). Interestingly, our data suggests that the deep habitats of the Gulf of Cadiz area harbors the highest diversity, with four ESUs observed out of four samples (Fig. 1). Currently there is one described *Plocamionida* species from the Antarctic (*Plocamionida gaussiana* (Hentschel, 1914), one from Washington (USA) (*Plocamionida lyoni* (Bakus, 1966), one from St Georges, Grenada (*Plocamionida topsenti* Burton, 1954) and none of them have been reported in the NE Atlantic. In addition, *P. gaussian*a and *P. lyoni* may lack proper short echinating acanthostyles, a morphological criteria shared by *P. topsenti* and the European *Plocamionida* species. Consequently, most of the ESUs investigated here might represent new taxa and our data show that the current number of *Plocamionida* species of the NE Atlantic waters may be underestimated. We did not encounter any individual that could be assigned to an *achelata* variety Topsent, 1928, but its existence may also indicate further diversity in the genus *Plocamionida*. A possible radiation of *Plocamionida* in the Gulf of Cadiz is suggested by our data, supporting the idea that ‘low latitude’ CWRs act as diversity hot-spots. Similarly, the bathyal Gulf of Cadiz area showed particularly high species diversity of marine hydroids [45], while an unprecedented number of unique evolutionary lineages of tubeworms was reported from the Gulf of Cadiz mud volcanoes [46]. Obviously, the sharp environmental discontinuities in temperature, pressure and nutrient richness (including silica concentration) in shallow water vs. deep-water coral reef habitats may have a great potential for sponge evolution. Our study adds to the growing evidence of genetically highly diverse CWRs and is expected to contribute to an improved understanding of the role of CWRs in the sustenance of sponge distributions along the coasts of Europe.

### Conclusion

Following an “integrative taxonomy” approach to study species from multiple, complementary perspectives [41], this study provides evidence that *P. grandichelata*, *P. tylotata*, *P. ambigua* and *P. microcionides* in the NE Atlantic are valid species, and suggests the existence of putatively new *Plocamionida* species. Those hypothetical species are now submitted to the filter of other approaches and further sampling followed by detailed phenotypic diagnostic analyses may support the observed molecular differences. New species are indeed important to consider for the protection of cold-water coral reefs, which are increasingly shown as reservoirs of biodiversity. On the other hand, the unexpected high level of *Plocamionida* biodiversity, found in the Gulf of Cadiz especially illustrates the problem of obtaining sufficient specimens in any one deep-sea sponge species from CWRs for phylogeographic studies. The higher amplification success and higher resolution power of I3M11 adds to the growing evidence that it may be a better COI partition than M1M6 to infer inter- and intraspecific diversity.

## Supporting Information

Table S1 ***Plocamionida*** **specimens analysed in the present study.** Information regarding the corresponding Evolutionary Significant Unit (ESU), sampling (code, localities with their abbreviation in parenthesis, sampling method, Field Number, voucher specimen, coordinates, depth), number of individuals studied (N) and number of different haplotypes (Nh) for each marker (COI M1M6 and I3M11 partition, 28S) is provided. Sampling location abbreviations are given in uppercase letters for deep-water samples (>50 m) and in lowercase letters for shallow-water samples.
(XLS)
